# Evaluation of the relationship between joint torque and angular velocity using a modified leg extension machine

**DOI:** 10.20407/fmj.2018-018

**Published:** 2019-09-25

**Authors:** Yoshikiyo Kanada, Hiroaki Sakurai, Yoshito Sugiura, Tomoaki Arai, Soichiro Koyama, Shigeo Tanabe

**Affiliations:** 1 Faculty of Rehabilitation, School of Health Sciences, Fujita Health University, Toyoake, Aichi, Japan; 2 Medical Corporation Howakai, Shima, Mie, Japan; 3 Department of Rehabilitation, Health Care Service Facility for the Aged, Shimahouwaen, Shima, Mie, Japan

**Keywords:** Joint torque, Angular velocity, One repetition maximum measurement, Leg extension machine, Muscle force

## Abstract

**Objective:**

When performing knee extension using a leg extension machine, the lower limb is pushed back in the direction in which knee flexion occurs in response to the freefall of the weight after maximal knee extension. Therefore, eccentric contractions of the knee extensors are needed, which may lead to cumulative fatigue of the extensors, consequently reducing the reliability of the knee extensor torque values. This study aimed to determine the relationship between joint torque and angular velocity in one repetition maximum (1RM) measurement for knee extension using a leg extension machine with and without a modification to prevent counter-rotation.

**Methods:**

Twenty-one healthy adult men (mean age: 27.7±5.4 years) participated in the study. A leg extension machine was modified to prevent counter-rotation due to the freefall of weights. The subjects performed knee extension using the modified leg extension machine, and the joint torque and angular velocity were calculated using two-dimensional analysis. A regression equation between these two factors was created to estimate the maximal isometric torque.

**Results:**

Both the joint torque and angular velocity tended to increase after modification of the leg extension machine, although these differences were not significant. Similarly, there were no significant post-modification changes in the estimated maximal isometric torque.

**Conclusions:**

Our results showed that the joint torque, angular velocity, and estimated maximal isometric torque remained unchanged after machine modification; thus, the modified leg extension machine may make it possible to produce the knee extensor torque more safely in 1RM measurement.

## Introduction

Resistance training is a general muscle training method used to increase the muscle mass and strength in individuals with reduced muscle strength.^1^ When setting the loads for resistance training, the maximal weight a subject can lift with one repetition (one repetition maximum; 1RM) is initially calculated to deduce the relative rates of this value (%1RM).^2–6^

In knee extension exercises, the 1RM tends to be estimated based on the relationship between the load and number of repetitions. Furthermore, as knee extension is an angular movement involving a single joint, it is recommended that the 1RM measurement should also involve measurements of the joint torque and angular velocity. Hill^7^ reported a curvilinear relationship between the angular velocity and loading level, as the former peaks when the latter is 0 kg, and the latter peaks when the former is 0 m/s. Jidovtseff et al.^8^ estimated the maximal isometric muscle force based on the load-velocity relationship using a bench press machine, and reported a significant correlation between the estimated values and the 1RM. Sekiguchi et al.^9^ also used a bench press machine to estimate the 1RM based on the load, number of repetitions, and velocity, and revealed a correlation between the estimated and actual measurement values. However, as these previous studies used wire-type velocity measurement devices, only the velocity of the vertical component was measurable, while it remained difficult to measure the angular velocity when executing monoarticular movements that are important in resistance training.

In addition to the joint torque produced with each weight lifted, the torques of the lower-leg, foot, and attachment load, and the inertia torques at the lower leg and foot must be considered. Sugiura et al.^10,11^ measured the angular velocity during knee extension using a leg extension machine and reported that minimum and maximum loading levels of 40%1RM and 150%1RM, respectively, are appropriate for the establishment of a favorable force-velocity relationship. They also achieved a high agreement rate between the estimated maximal isometric extensor torque based on the force-velocity relationship and the actual measurement values representing such a torque, obtained using an isokinetic exercise machine (intraclass coefficient (ICC)_(2,__1)_ 0.86–0.87).^12^

When performing knee extension using a leg extension machine, the lower limb is pushed back in the direction in which knee flexion occurs in response to the freefall of the weight after maximal knee extension. Therefore, eccentric contractions of the knee extensors are needed to maintain the weight at the final point of lifting. Such eccentric contractions, which are unnecessary in the setting of loads for 1RM measurement, may lead to cumulative fatigue of the knee extensors, consequently reducing the reliability of the knee extensor torque values. To resolve this problem, we modified a leg extension machine to minimize the influence of muscle fatigue due to repeated eccentric concentrations of the knee extensors on 1RM measurement, and reported its effect in our previous study.^13^ In the present study, we did not examine the angular acceleration-dependent torque values. The objective of the present study was to determine the relationship between the joint torque and angular velocity when performing knee extension through comparison of the values before and after modification of a leg extension machine.

## Methods

### Subjects

The study involved 21 healthy men without orthopedic disorders of the knee joint (mean age: 27.7±5.4 years; height: 170.5±5.6 cm; bodyweight: 66.3±12.8 kg). The exclusion criteria^14,15^ were: 1) individuals with movement restrictions detected by their family physicians, 2) difficulty in understanding the instructions on how to carry out the motor tasks, 3) pain that may lead to the need for discontinuation of the motor tasks, 4) rapidly progressing, acute, or unstable chronic diseases, 5) history of hypertension or tachycardia, and 6) orthopedic diseases of the knee joint.

### Ethical approval

The study was approved by the ethics committees of the study corporative body (approval number: 16-005) and university (HM16-087). Furthermore, the subjects were provided with written and oral explanations of the study objective, and their written informed consent was obtained prior to study commencement.

### Measurement methods

The study protocol is shown in Table 1. The joint torque and angular velocity when performing knee extension were measured before and after modification of the leg extension machine (NR-S; Senoh Corporation, Japan) with a ratchet (R-160; Sanwa Conveyer Co., Ltd., Japan) to prevent the freefall of weights that induced counter-rotation. The ratchet was firmly welded to the knee rotation axis of the leg extension machine. During measurement, the machine was used under two different conditions: 1) with the ratchet pawl lifted to keep the machine unfixed during measurement (before modification), and 2) with the ratchet pawl set down (after modification). The final point of lifting to determine the knee extension range of motion for the 1RM was measured using a stadiometer (Seca-213; Seca Nihon Co., Ltd., Japan).

**Figure 1:**
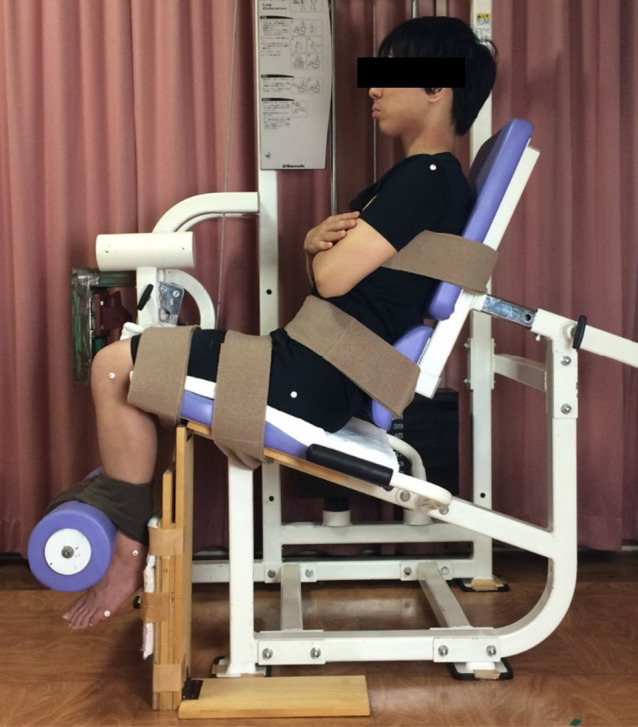
Photograph showing the testing setup.

Prior to 1RM measurement, a 10-minute ergometer workout was conducted as a warm-up at an appropriate intensity level as assessed via the Borg Scale. Subsequently, each subject sat on the leg extension machine with their chest, pelvis, proximal and distal parts of the thigh, and foot immobilized using belts. For the measurement, the left leg was used, while the right leg was kept hanging down. The initial position comprised a hip flexion angle of 70° and a knee flexion angle of 100°, with both upper limbs kept crossed in front of the chest (Figure 1).^13^ While limiting knee extension to maximum-effort concentric contractions, the measurement was performed at a knee angle of 90° to the final point of lifting.

When measuring the 1RM, maximal voluntary knee extension without loading was performed five times to enable the calculation of a mean value in cm (values were rounded down to one decimal place) as the final point of lifting. Subsequently, to predict the weight that would be initially lifted, knee extension was performed to the final point of lifting at a loading level assessed via the Borg Scale. The loading level was adjusted at intervals of 0.5 kg until the maximum weight each subject could lift was determined.^10–12^ When it was possible for the subject to lift the weight at least once during any of the three measurement sessions at the same loading level, the weight was increased. The subjects were given 30-second and 3-minute rests between measurement sessions and load adjustments, respectively. For the velocity measurements, the loading level for the 1RM was defined as 100%. Based on this, six patterns of loading (40%1RM, 60%1RM, 90%1RM, 100%1RM, 130%1RM, and 150%1RM) were applied three times each.

Two-dimensional movements were filmed using a high-speed video camera (GC-P100; JVC, Japan) at a rate of 240 frames per second, with markers attached to the left acromion and the following parts of the left leg: greater trochanter, knee joint cleft, lateral malleolus, and head of the fifth metatarsal bone. The center of the high-speed video camera lens was level with the marker attached to the knee joint cleft. The distance between the camera and each subject was 1,700 mm. The high-speed video camera was placed so that the marker attached to the knee joint cleft was vertically and horizontally evenly displayed in the image plane at the maximum zoom level.

The obtained visual data were converted into continuous still images at intervals of 1/240 seconds using the dynamic image editing software GOM PLAYER (from a knee flexion angle of 90° to the final point of lifting). Image J image instrumentation software was used to measure the coordinates of each articulation point in pixel values and convert these into meter values. Furthermore, these coordinates were incorporated into a dynamic model to calculate the joint torque and angle. Based on the lower-leg, foot, and attachment load torques, and the inertia torques at the lower leg and foot in addition to the joint torque produced with each weight lifted, the following kinematic equation for knee extension was created:

$$Tk=M\times g\times \ell_{1}+\mathrm{BW}\times \mathrm{0.0725}g\times \sin\theta \times \ell_{2}+\mathrm{0.158}\times \ddot{\theta }$$

**T*****k***** (N·m)**: knee extensor torque

***M***** (kg)**: load

***G***** (9.8 m·s^–2^)**: gravitational acceleration

**ℓ_1_ (m)**: distance from the knee joint cleft to the load point

***BW***** (kg)**: bodyweight

**0.0725**: ratio of the left lower leg and foot to the bodyweight

***θ***: knee flexion angle (angle of knee extension in a posture with the lower leg hanging down, with complete knee extension defined as 0°)

**ℓ_2_ (m)**: distance from the knee joint cleft to the synthesized center of gravity between the lower leg and foot

**0.158**: drag coefficient of the left lower leg and foot

$\ddot{\theta }$ **(rad·s^–2^)**: angular acceleration

### Statistical analysis

The angular velocity was calculated based on the trial time, by dividing the number of bitmap files from a knee angle of 90° to the final point of lifting by 240 frames. Of the three measurements of knee extension with each of the six loads, the peak angular velocity was adopted as the angular velocity. The angular velocities before and after modification were compared using the Mann-Whitney U test.

In each loading pattern, the mean and maximum knee extensor torque values obtained in the measurement with the peak angular velocity were adopted as the mean and maximum torques, respectively; the latter was the value at a point between the knee angle of 90° and the final point of lifting, at which the maximum torque value was obtained. The mean and maximum torque values before and after modification were compared using the Mann-Whitney U test. Additionally, the correlations between the joint torque and angular velocity in the following four combinations were examined using Spearman’s rank correlation coefficient: 1) average torque+angular velocity before modification, 2) maximum torque+angular velocity before modification, 3) average torque+angular velocity after modification, and 4) maximum torque+angular velocity after modification.

To clarify the relationship between the joint torque and angular velocity with each load in each subject, regression analysis was performed with the two factors as dependent and independent variables, respectively. Furthermore, an angular velocity of 0 d·s^–1^ (the point at which the maximal isometric extensor torque was obtained) was incorporated into the created regression equation to estimate the maximal isometric extensor torque. The estimated maximal isometric extensor torque values were compared using the Mann-Whitney U test. The agreement between the estimated maximal isometric extensor torques before and after modification was examined by calculating the ICC and performing the Bland-Altman analysis.

All statistical processing was performed using SPSS Statistics version 21 software (IBM, Armonk, NY, USA), with the significance level set at 5%.

## Results

**Figure 2:**
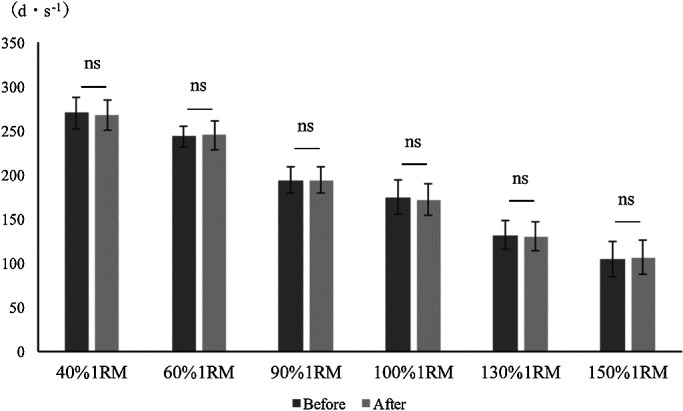
Comparison of peak angular velocity before and after modification of the leg extension machine. Mann-Whitney U test. *p<0.05 There was no significant difference in peak angular velocity before versus after modification.

**Figure 3:**
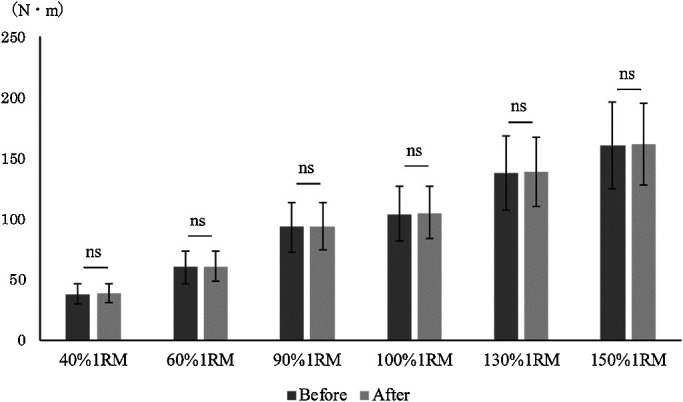
Comparison of average torque before and after modification of the leg extension machine. Mann-Whitney U test. *p<0.05 There was no significant difference in average torque before versus after modification.

**Figure 4:**
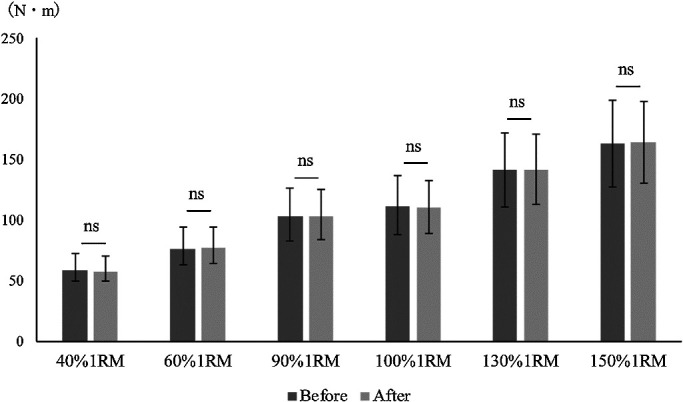
Comparison of maximum torque before and after modification of the leg extension machine. Mann-Whitney U test. *p<0.05 There was no significant difference in maximum torque before versus after modification.

**Figure 5:**
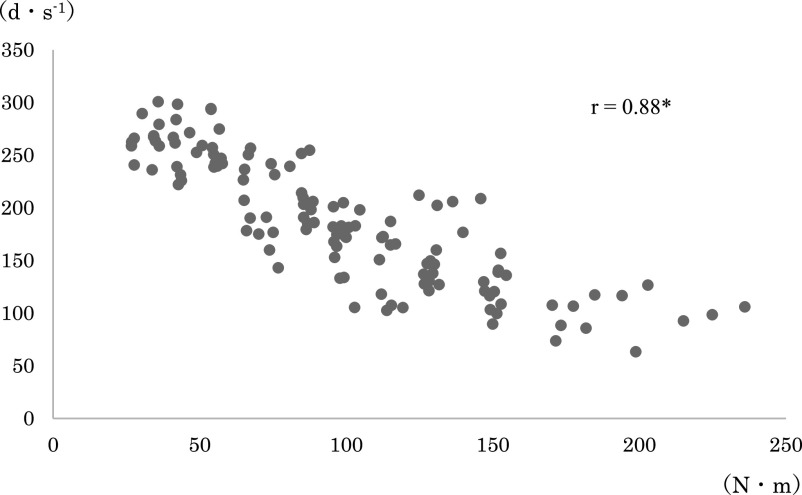
Correlation coefficient between angular velocity and average torque before modification of the leg extension machine. Spearman’s rank correlation coefficient. *p<0.01

**Figure 6:**
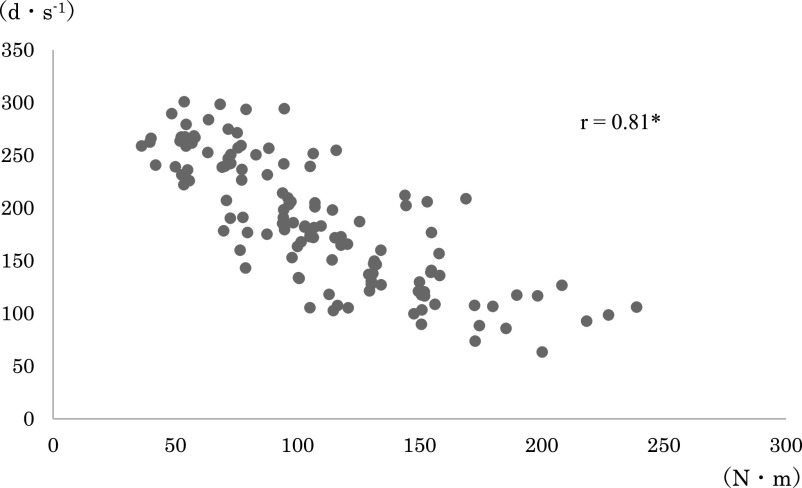
Correlation coefficient between angular velocity and maximum torque before modification of the leg extension machine. Spearman’s rank correlation coefficient. *p<0.01 The correlation was weaker than that in Figure 5.

**Figure 7:**
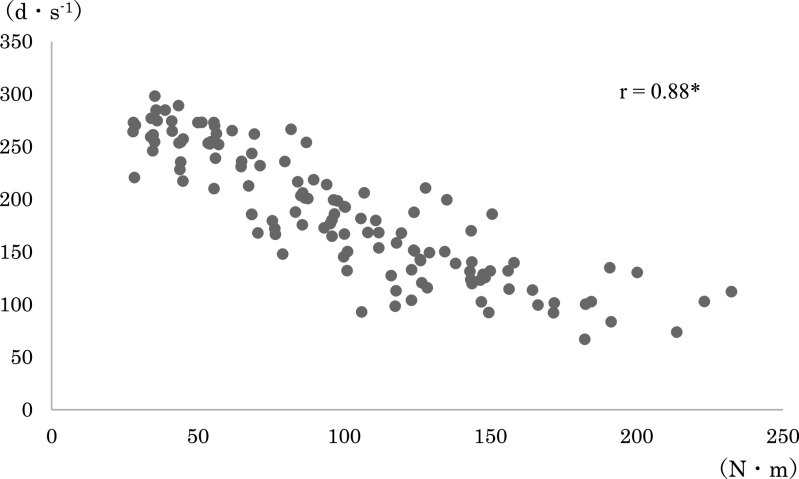
Correlation coefficient between angular velocity and average torque after modification of the leg extension machine. Spearman’s rank correlation coefficient. *p<0.01

**Figure 8:**
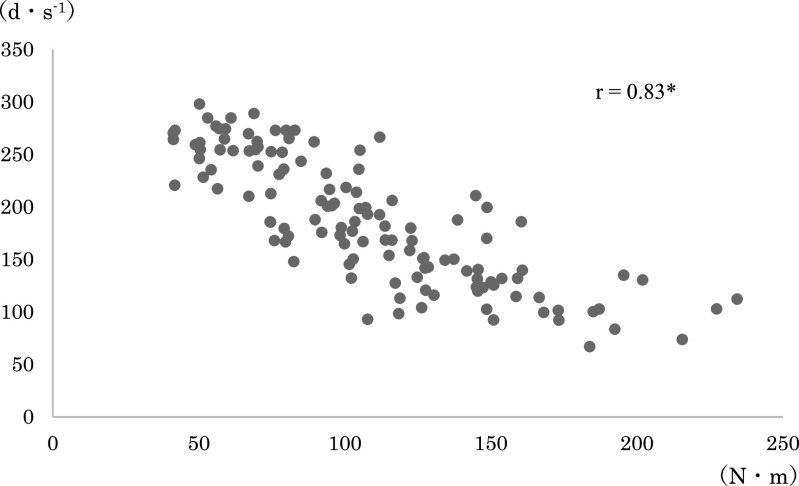
Correlation coefficient between angular velocity and maximum torque after modification of the leg extension machine. Spearman’s rank correlation coefficient. *p<0.01 The correlation was weaker than that in Figure 7.

**Figure 9:**
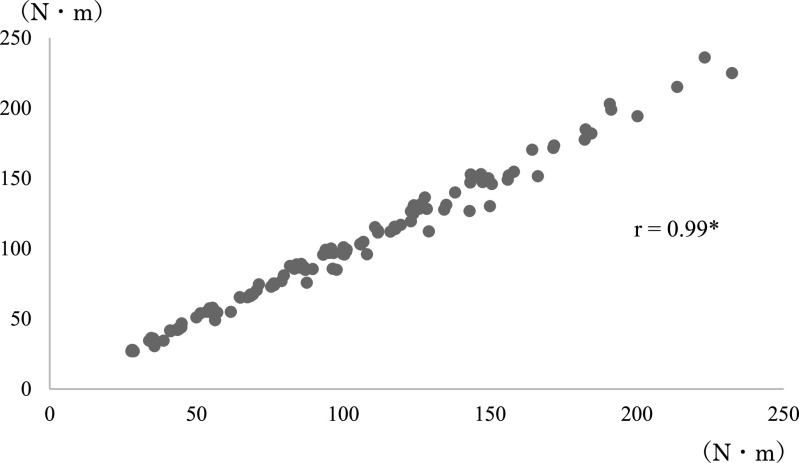
Correlation coefficient for average torque before and after modification of the leg extension machine. Spearman’s rank correlation coefficient. *p<0.01. The average torque was strongly correlated before and after modification.

**Figure 10:**
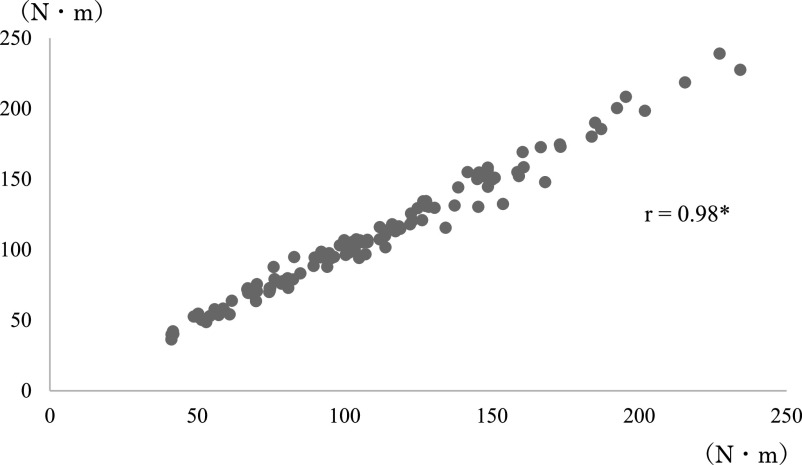
Correlation coefficient for maximum torque before and after modification of the leg extension machine. Spearman’s rank correlation coefficient. *p<0.01 The maximum torque was strongly correlated before and after modification.

The angular velocity did not significantly vary before versus after modification of the leg extension machine (Figure 2). Similarly, there were no significant differences in the mean or maximum torque value between the two conditions using any loading pattern (Figures 3 and 4). The correlation coefficients between the joint torque and angular velocity in each combination were: r=0.88 for average torque+angular velocity before modification; r=0.81 for maximum torque+angular velocity before modification; r=0.88 for average torque+angular velocity after modification; and r=0.83 for maximum torque+angular velocity after modification (Figures 5, 6, 7, 8, 9, 10).

**Figure 11:**
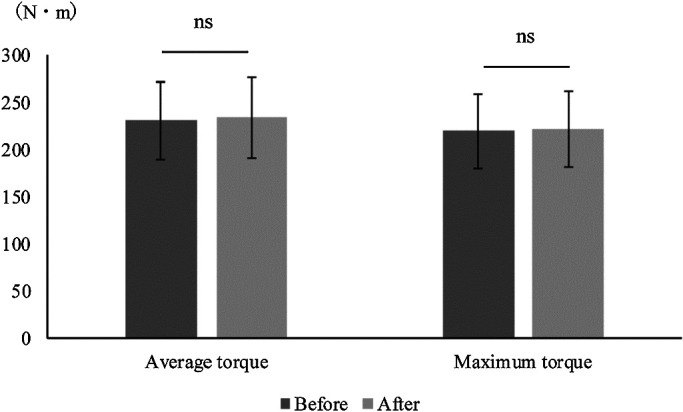
Comparison of isometric maximum torque estimates. Mann-Whitney U test. *p<0.05

**Figure 12:**
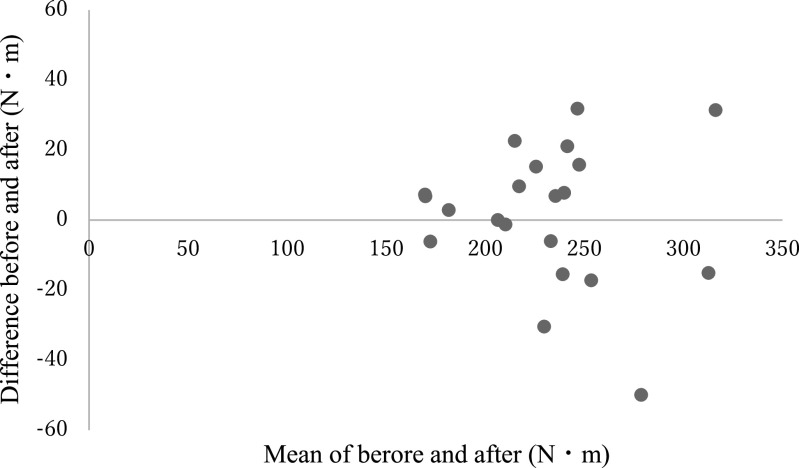
Bland-Altman graph showing each of the estimated maximal isometric torques before and after modification of the leg extension machine (Test 1: average angular velocity and average torque).

**Figure 13:**
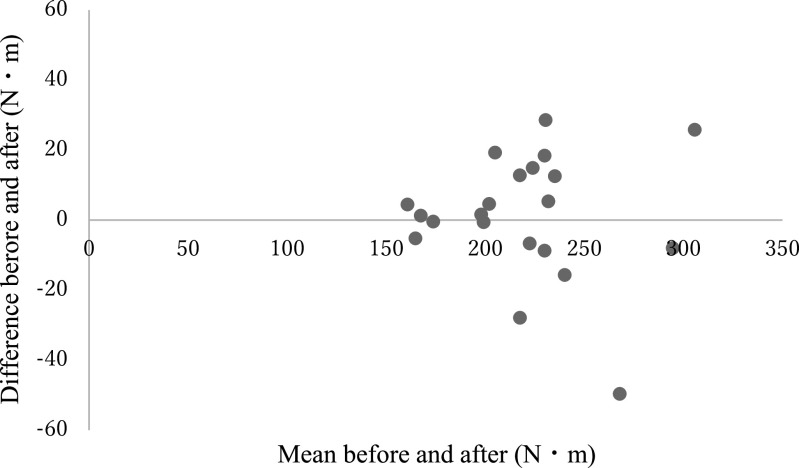
Bland-Altman graph showing each of the estimated maximal isometric torques before and after modification of the leg extension machine (Test 2: average angular velocity and maximum torque).

Regarding the relationship between the joint torque and angular velocity, no significant differences were observed in the estimated maximal isometric extensor torque before versus after modification of the leg extension machine (Figure 11). The reliabilities of the estimated maximal isometric extensor torque in each combination before/after modification were: ICC_(2,1)_=0.93 for average torque+angular velocity, and ICC_(2,1)_=0.93 for maximum torque+angular velocity. The Bland-Altman analysis did not reveal a fixed or proportional bias (Table 2, Figures 12 and 13).

## Discussion

The present study compared the joint torque and angular velocity in healthy men before versus after modification of a leg extension machine with a ratchet. It also examined the relationship between these two factors (force-velocity relationship). There were no significant differences in the joint torque or angular velocity before versus after modification of the machine. There have been few reports confirming the force-velocity relationship in human isotonic movements, and data regarding knee extension, representing the lower-limb muscle force, have not been fully examined. The quadriceps femoris is the agonist for knee extension, and its force is reportedly strongly correlated with the performance of activities of daily life.^16–18^

Although there were no significant differences in the angular velocity before versus after machine modification, the value slightly increased after modification using four loading patterns (40%1RM, 60%1RM, 90%1RM, and 100%1RM). Regarding the force-velocity relationship, the self-adjustment range of the angular velocity is reportedly wider at lesser loading levels, and therefore the angular velocity can be purposefully adjusted.^10^ At loading levels of 40 to 100%1RM, similarly to the case of 1RM values, the use of the modified machine reduces the number of tasks requiring the allocation of attention to “executing rapid movements”. This allows subjects to concentrate on concentric contraction for knee extension, explaining the large angular velocity achieved as a result of improved performance in the present study. In contrast, at loading levels of 130 to 150%1RM, the self-adjustment mechanism of the angular velocity does not work due to the excessive loads. Consequently, the value may become force-dependent, rather than velocity-dependent. In short, when executing movements at larger loading levels, the influence of the loads may have been greater than that of attention allocation to the simultaneous performance of multiple tasks, resulting in no changes in the angular velocity.

The relationship between the joint torque and angular velocity was examined in six loading patterns from 40% to 150%1RM applied before and after modification of the leg extension machine. The angular velocity was strongly correlated with the mean and maximum torques both before and after modification (Figures 5–10). A correlation between the joint torque and angular velocity was also reported in previous studies, with high correlation coefficients of 0.95 and 0.92 for average torque+angular velocity and maximum torque+angular velocity, respectively.^11^ The combination of average torque+angular velocity also showed a strong correlation in the present study. The correlation between the joint torque and angular velocity was stronger after modification of the leg extension machine than that before it.

The maximal isometric muscle force is widely used as an index to evaluate muscle contraction and determine training intensity levels.^19^ However, as it requires high exercise intensity, there are concerns that this may increase the risks of hypertension and muscle tendon injury.^20,21^ In the present study, the maximal isometric torque when performing knee extension at loading levels of 40 to 150%1RM was estimated based on the relationship between the joint torque and angular velocity; the value before modification of the leg extension machine was 219.4 to 231.4 N·m, while the value after modification had increased to 220.6 to 234.0 N·m. The use of the modified leg extension machine may have reduced fatigue and the number of tasks required to be performed simultaneously, consequently promoting concentric contraction for knee extension. Examination of the ICCs before and after modification in various combinations showed that favorable agreement was achieved. The combination of average torque+angular velocity showed the strongest correlation, but the agreement rate for the estimated maximal isometric torque was similarly high in this combination and in maximum torque+angular velocity. Average torque+angular velocity also showed a strong correlation in a previous study.^11^ However, when focusing on the agreement rate between the estimated and actual maximal isometric extensor torque values, maximum torque+angular velocity showed the highest rate.^11^ Having measured these factors at maximum effort, the authors concluded that it was most appropriate to estimate the maximal isometric extensor torque in maximum torque+angular velocity.^11^ In the present study, which also measured maximum-effort contraction, this combination may have been the most appropriate to estimate the maximal isometric torque.

In future, it may be necessary to use our load-determining method for muscle strengthening and confirm its effect in the clinical setting. As we only analyzed the knee extensor force of healthy men in their twenties and thirties, the versatility of the method should be extensively examined in similar studies involving various age- and sex-based groups, as well as other muscles. Moreover, it should also be confirmed that the maximal isometric torque estimated using the modified leg extension machine is in agreement with actual measurement values obtained with isokinetic exercise devices.

## Conclusion

In the present study, the estimated maximal isometric torque for muscle strengthening was compared before and after machine modification. In future, we aim to verify the coincidence rate between the calculated estimate and the actual value measured using constant velocity exercise equipment. Based on our study, it can be expected that safe muscle load settings can be easily applied in the fields of geriatric care and health promotion.

## Figures and Tables

**Table1:** Study protocol

Step 1.	One repetition maximum (1RM) measurement for knee extension
Step 2.	Video capture of the two-dimensional movements
Step 3.	Calculation of angular velocity
Step 4.	Calculation of average and maximum torques
Step 5.	Estimation of isometric peak torque in knee extension

Steps 1–5 were performed both before and after modification of the leg extension machine

**Table2:** Reliability of each estimate of isometric maximum torque before and after modification of the leg extension machine

Test	Mean difference (mean±SD)	ICC_(2,1)_	Bland-Altman analysis		SEM	MDC_95_	LOA
			Fixed bias (95% CI)	Proportional bias (Pearson)			
Test 1	1.8±19.9	0.89	–10.8 to 7.3	r=0.10 *p*=0.68	14.1	39	–24.7 to 21.2
Test 2	1.2±18.0	0.9	–9.4 to 7.0	r=0.06 *p*=0.81	12.7	35.3	–22.0 to 19.5

Test 1: angular velocity and average torque; Test 2: angular velocity and maximum torque; Mean difference: mean and standard deviation of the difference between the before-modification estimate and the post-modification estimate; ICC: intraclass correlation coefficients; 95% CI: 95% confidence interval, Pearson: Pearson product-moment correlation coefficient; SEM: standard error of measurement; MDC95: minimal detectable change; LOA: limits of agreement
